# CAR-T-Derived Extracellular Vesicles: A Promising Development of CAR-T Anti-Tumor Therapy

**DOI:** 10.3390/cancers15041052

**Published:** 2023-02-07

**Authors:** Sara Pagotto, Pasquale Simeone, Davide Brocco, Giulia Catitti, Domenico De Bellis, Simone Vespa, Natalia Di Pietro, Lisa Marinelli, Antonio Di Stefano, Serena Veschi, Laura De Lellis, Fabio Verginelli, Francesco Kaitsas, Manuela Iezzi, Assunta Pandolfi, Rosa Visone, Nicola Tinari, Ignazio Caruana, Mauro Di Ianni, Alessandro Cama, Paola Lanuti, Rosalba Florio

**Affiliations:** 1 Department of Medical, Oral and Biotechnological Sciences, “G.d’Annunzio” University of Chieti-Pescara, 66100 Chieti, Italy; 2 Center for Advanced Studies and Technology (CAST), “G.d’Annunzio” University of Chieti-Pescara, 66100 Chieti, Italy; 3 Department of Medicine and Aging Sciences, “G.d’Annunzio” University of Chieti-Pescara, 66100 Chieti, Italy; 4 Department of Pharmacy, “G.d’Annunzio” University of Chieti-Pescara, 66100 Chieti, Italy; 5 Sacred Heart Catholic University, 00168 Rome, Italy; 6 Department of Neuroscience, Imaging and Clinical Sciences, “G.d’Annunzio” University of Chieti-Pescara, 66100 Chieti, Italy; 7 Department of Pediatric Haemaology, Oncology and Stem Cell Transplantation, University Hospital Würzburg, 97080 Würzburg, Germany

**Keywords:** extracellular vesicles, CAR-T cells, tumors, anti-tumor agents

## Abstract

**Simple Summary:**

In this review we aim to address the potential of extracellular vesicles stemming from chimeric antigen receptor T (CAR-T) lymphocytes as therapeutic agents in tumors. We underlined how CAR-T-lymphocytes, representing one of the new frontiers of immunotherapy for the fight against refractory neoplastic diseases, demonstrated their potential effectiveness in cancer. However, the presence of physical barriers that prevent the entry of CAR-T and other immune effector cells, the hostile microenvironment that hampers persistence and activity of immune cells, as well as tumor heterogeneity resulted in their variable or low efficacy against solid tumors. The application of CAR-T-derived extracellular vesicles as therapeutic agents may improve the homing of CAR-T effector functions through their facilitated diffusion within solid tumors and at the same time might circumvent some of the adverse effects that are induced by the cellular counterpart.

**Abstract:**

Extracellular vesicles (EVs) are a heterogenous population of plasma membrane-surrounded particles that are released in the extracellular milieu by almost all types of living cells. EVs are key players in intercellular crosstalk, both locally and systemically, given that they deliver their cargoes (consisting of proteins, lipids, mRNAs, miRNAs, and DNA fragments) to target cells, crossing biological barriers. Those mechanisms further trigger a wide range of biological responses. Interestingly, EV phenotypes and cargoes and, therefore, their functions, stem from their specific parental cells. For these reasons, EVs have been proposed as promising candidates for EV-based, cell-free therapies. One of the new frontiers of cell-based immunotherapy for the fight against refractory neoplastic diseases is represented by genetically engineered chimeric antigen receptor T (CAR-T) lymphocytes, which in recent years have demonstrated their effectiveness by reaching commercialization and clinical application for some neoplastic diseases. CAR-T-derived EVs represent a recent promising development of CAR-T immunotherapy approaches. This crosscutting innovative strategy is designed to exploit the advantages of genetically engineered cell-based immunotherapy together with those of cell-free EVs, which in principle might be safer and more efficient in crossing biological and tumor-associated barriers. In this review, we underlined the potential of CAR-T-derived EVs as therapeutic agents in tumors.

## 1. Introduction

Communication has a central role in all biological functions and its evolutionary history dates to the earliest forms of living organisms on Earth. One of the mechanisms that is used by cells to communicate is based on the release of membrane-surrounded particles, named extracellular vesicles (EVs), stemming from almost all living prokaryotic and eukaryotic cell types. It was shown that their releasing processes have been conserved throughout evolution [1], and recent studies underlined their role as biological carriers of messages delivered through biological fluids and acting as key players of the intercellular crosstalk [2,3,4]. EVs carry proteins, lipids, and different types of RNAs and DNAs [5,6,7,8], which are transferred to target cells, influencing their functions [9]. It is also known that EVs are distributed in all body fluids, such as peripheral blood, saliva, tears, cerebrospinal fluid, and urine [10,11,12,13,14,15,16,17]. In the last decades, EVs have been involved in many physiological and pathological processes, therefore, attracting considerable attention both as disease biomarkers and as drug delivery vehicles [18,19,20,21,22,23].

It was demonstrated that EVs can cross different biological barriers, such as the blood-brain barrier (BBB) and tumor physical barriers [1]. Furthermore, compared to other nanoparticles, such as synthetic particles, nanomaterials, or lipid transfection agents, EVs show a low grade of cytotoxicity and immunogenicity [24,25,26,27] and, therefore, have great potential as drug delivery agents for cell-free therapies in several contexts [28,29,30].

Among genetically engineered cell-based therapies, chimeric antigen receptor (CAR)-T cells emerged as an innovative and powerful therapeutic tool against malignant cells [31]. CAR-T cells are genetically engineered T cells expressing recombinant receptors consisting of an extracellular target-recognition domain, generally arising from single-chain variable fragments (scFv), a transmembrane domain, and intracellular tyrosine-based activation motifs (ITAMS). By their extracellular domain, CAR-T cells recognize specific molecules on tumor cell membranes, while the intracellular domain triggers the T cell receptor (TCR) signaling activation, causing the death of target cells without the involvement of the major histocompatibility complex (MHC) molecules [32,33,34].

Besides the successful application of CAR-T cells in the treatment of B cell malignancies, their efficacy in other neoplastic diseases and largely in solid tumors is still limited. Several studies have underlined how the immunosuppressive tumor microenvironment (TME) that is present in these tumors negatively impacts CAR-T cell activity and long-term persistence, inducing their differentiation, exhaustion, and/or anergy [35,36,37,38]. The TME also represents a physical barrier that is difficult to penetrate by lymphocytes and CAR-T cells, reducing their anti-tumor activities [39,40]. As mentioned above, EVs do not present this limitation, being able to cross different biological barriers, including those that are present in tumors. Therefore, it is conceivable that CAR-T-derived EVs may circumvent the barriers that are encountered by cell-based approaches and may represent promising therapeutic agents in the treatment of refractory malignancies.

This review describes the results and the impact of CAR-T cells in the treatment of several malignancies, then it examines the EV biological functions and focuses on the potential of CAR-T-derived EVs as therapeutic agents for both hematological and solid tumors.

## 2. Chimeric Antigen Receptor (CAR)-T Cell, Structure, and Evolution

**Figure 1:**
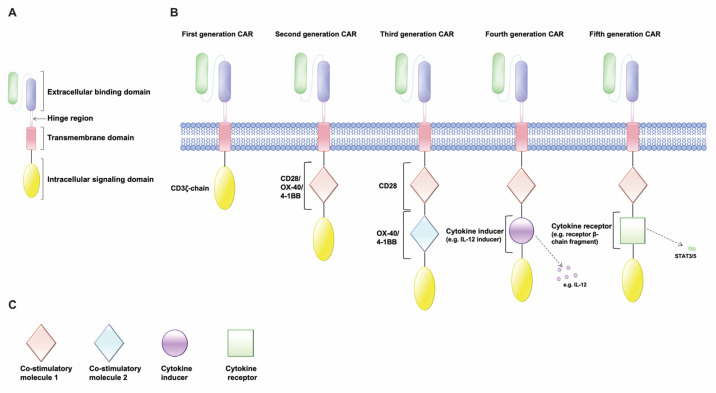
Structure and evolution of CAR-T cells. (**A**) The CAR contains an extracellular binding domain (usually in the form of scFv), a hinge region, a transmembrane domain, and an intracellular signaling domain. (**B**) The first generation of CARs has only a single transduction intracellular domain such as the CD3ζ-chain, while the second generation includes the addition of one co-stimulatory molecule such as CD28, OX-40 (CD134), and 4-1BB (CD137). The third CAR generation, instead, is equipped with two different co-stimulatory molecules; while the fourth generation contains usually one costimulatory molecule and a transcription factor that is capable of inducing the production of specific cytokines (e.g., IL-12). Lastly, the fifth CAR generation has been developed based on the second CAR generation with the addition of an extra intracellular domain of cytokine receptors with a binding site for transcription factors, such as STAT-3/5. (**C**) Different intracellular domains of CAR-T cells.

CARs are synthetic chimeric proteins that typically consist of four major components: (1) an extracellular target-binding domain, in most cases in the form of a scFv, which is derived from a monoclonal antibody that confers target antigen specificity [41]; (2) a hinge region, that provides flexibility to allow the antigen-binding domain to access the targeted epitope [42]; (3) a transmembrane domain that is derived from natural proteins including CD3ζ, CD4, CD8α, or CD28, which is an ɑ-helix within cell membranes that anchors the transgenic protein to the cellular membrane linking the extracellular antigen-binding domain to the intracellular cytoplasmic domain [43]; and (4) an intracellular signaling domain, which generally consists of one or more costimulatory domains fused to a CD3ζ or a γ chain of the high-affinity IgE Fc receptor (FcεRI) (Figure 1A). When expressed by T lymphocytes, the scFv determines the CAR antigen specificity and allows T cells to engage the antigen that is expressed by tumor cells in an MHC-independent manner. After binding to the target, cross-linked CAR molecules activate the endo-domain signaling, thus inducing the lysis of the engaged target cells through granzyme-B and perforin pathways [44,45]. Although their fundamental modular structure remained similar since their inception in the late 1980s [46], the improvement of the design of CAR-T cells has been the focus of recent years, resulting in five distinct generations according to the shape of their intracellular domain (Figure 1B,C).

The first-generation of CARs contained a single CD3ζ-chain intracellular domain, however this generation of CARs revealed two major drawbacks: the inability to achieve a high enough level of antitumor activity (insufficient IL-2 production) and a short lifespan for their lack of proliferation (Figure 1B) [47].

The second generation of CAR-T cells displayed a better immune response by leveraging the power of the co-stimulatory signaling domain (molecules such as CD28, CD134 or OX-40, CD137 or 4-1BB, etc., which are fused with CD3ζ), enhancing cytokine release, cytotoxicity, and their in vivo half-lives (Figure 1B) [48,49]. Subsequently, the third generation of CARs has been designed by combining multiple co-stimulatory signaling to improve the function of CAR-T cells (CD3ζ-CD28-OX40 or CD3ζ-CD28-41BB); however, the enhanced efficacy in comparison to second-generation CAR-T cells is still unclear (Figure 1B) [50,51,52]. The fourth generation of CARs, instead, is a second/third-generation construct including a T cell response element similar to the nuclear factor of the activated T cell (NFAT) that is capable of inducing the production of cytokines as IL-12, IL-13, and GM-CSF upon activation, thus providing benefits to T cells or the other elements of the immune system (Figure 1B) [40,53,54]. The fifth generation of CAR-T cells contains a supplementary intracellular domain of cytokine receptors (e.g., receptor β-chain fragment) with a binding site for the transcription factors such as STAT-3/5 (Figure 1B). The antigen-specific activation of this receptor simultaneously triggers three synergistic signals: the CAR-T cells activation, the memory T cell generation, and the reactivation-stimulation of the immune system [55].

Nevertheless, it must be underlined that CAR-T cell products (already used or under testing) are predominant autologous. Recently, to overcome the limitation that was observed with autologous and allogeneic CAR-T cell products, universal CAR-T (UCAR-T) cells have been developed. UCAR-T cells share the same killing mechanism as CAR-T, but they are characterized by different cell manufacturing processes and a better applicability [56]. UCAR-T cells are produced by a method that allows them to effectively abolish graft-versus-host disease (GvHD) by genetically disrupting the HLA Class I loci of the allogeneic T cells and/or TCR gene, by targeting genomic sequences in the constant regions of the endogenous α, or β subunits of the TCR or disrupting HLA-A locus of MHC gene complex. In this way, T cells do not recognize allogeneic antigens. For these purposes, zinc finger nuclease [57,58], transcription activator-like effector nuclease (TALEN) [59], and the CRISPR/Cas9 system [60,61,62] are the applied gene-editing techniques for obtaining UCAR-T cells. Many preclinical and clinical trials using UCAR-T have been activated worldwide [56,63]. Many of these studies produced UCAR-T that were directed against hematological malignancies, and CD19 is the most used target, even if CD5, CD7, CD20, CD22, and BCMA have been also used for these purposes [56,64]. Furthermore, GD2, NK group 2 member D ligand (NKG2DL) and mesothelin have also been tested for solid tumors [56,65,66].

## 3. Overview of CAR-T Cell Therapies in Hematological Malignancies

CAR-T cells represent one of the most revolutionary therapies for certain hematological malignancies, such as B cell acute lymphoblastic leukemia (B-ALL), diffuse large B cell lymphoma (DLBCL), multiple myeloma (MM), mantle cell lymphoma (MCL), and follicular lymphoma (FL). The US Food and Drug Administration (FDA) approved the first anti-CD19 CAR-T cell therapy for B cell malignancies in 2017. So far, another five CAR-T cell therapies have been approved, four of them targeting the CD19 antigen, and two targeting the B cell maturation antigen (BCMA) [67,68,69]. Although CAR-T cell therapies achieved outstanding outcomes in B-cell malignancies, disease relapse can be observed after treatment. The mechanisms that are responsible for CAR-T resistance and thus for disease recurrence include the antigen loss [70], the inadvertent transduction of tumor blast with CAR19 [71], and T cell exhaustion [38,72]. The most commonly observed adverse effects after CAR-T treatment are cytokine release syndrome (CRS) and immune effector cell-associated neurotoxicity syndrome (ICANS) whose management has been recently standardized [73].

### 3.1. Anti CD19-Approved CAR-T Cell Therapies

The first CAR-T cell product that was approved by the FDA (August 2017) was tisagenlecleucel (Kymriah™). It is an autologous CD19-targeted CAR-T cell product that is used for the pediatric and young adult treatment of relapsed/refractory (R/R) B-ALL [74]. More recently (May 2022), tisagenlecleucel was also approved by the FDA for the treatment of adult patients with R/R follicular lymphoma after two or more lines of systemic therapies [75]. FDA approval was based on the results of the global Phase 2 ELARA trial (NCT03568461), involving 94 patients. The results of this study showed an overall response rate (ORR) of 86%, even if complete responses occurred in 49% of patients (Grade ≥ 3, in 0%) neurological events in 37.1% (Grade ≥ 3, in 3%) and ICANS in 4.1% (Grade ≥ 3, in 1%). No treatment-related deaths were registered [75].

The axicabtagene ciloleucel (Yescarta™) is a cell product that is based on CD19 CAR-T cells and it was FDA approved in 2017 for the treatment of adult patients with DLBCL and with primary mediastinal large B cell lymphoma. Its approval was based on the results of the ZUMA-1 multicenter trial [67]. In this study, 111 patients received CD19 CAR-T cells; the ORR was 82%, and the complete response rate was 54%. Notably, cytokine release syndrome occurred in 90% of the treated patients (Grade ≥ 3, in 9%), while neurologic toxicity was evidenced in 78% of cases (Grade ≥ 3, in 25%).

In 2020, the FDA approved the use of brexucabtagene autoleucel (Tecartus™), a third generation CAR-T cell drug (CD19/FMC63), for treating R/R MCL patients [67]. Later, in October 2021, the FDA approved the use of Tecartus™, also for the treatment of adult patients (older than 26) that were affected by R/R B-ALL. In the pivotal Phase 2 ZUMA-3 trial (NCT02614066), 39 out of 55 patients (71%) had complete remission or complete remission with incomplete hematologic recovery, with 31 (56%) patients reaching complete remission. The most common adverse events of Grade 3 or higher were anemia (49%) and pyrexia (36%). CRS of Grade 3 or higher occurred in 13 (24%) patients, and neurological events of Grade 3 or higher occurred in 14 (25%) patients [76].

In 2021 (February), lisocabtagene maraleucel (liso-cel, Breyanzi™), a cell product based on CD19 CAR-T cells equipped with a 4-1BB costimulatory domain, was also approved by the FDA for R/R large B cell lymphoma treatment [77]. The approval was based on the results of the TRANSCEND NHL001 (NCT02631044) trial involving 268 patients [78]. The ORR was 73%, with the occurrence of CRS (42% of patients, Grade ≥ 3 in 4%) of treated patients. Neurologic toxicities occurred in 35% (95/268) of patients (Grade ≥ 3 in 12%, 31/268 patients).

### 3.2. Anti-BCMA Approved CAR-T Cell Therapies

Among the anti BCMA CAR-T therapies that have been approved by the FDA for hematological malignancies, the idecabtagene vicleucel (Abecma™) must be cited. It is the first anti-BCMA CAR-T cell therapy that was approved by the FDA (March 2021), used for the treatment of R/R MM after fourth-line or more therapies [69,79]. It was reported that, among patients treated with idecabtagene vicleucel (*n* = 128), 73% of them achieved an ORR, while for 28% of them a stringent complete response rate (sCR) was observed. Of note, CRS occurred in 85% of treated patients with mainly Grade 1–2. Neurotoxicity was observed in 18% of patients (3% Grade 3; no Grade 4 or 5). Recently, the results of a two-years follow-up of the administration of ciltacabtagene autoleucel (Carvykti™), a differentiated CAR-T therapy with two BCMA-targeting single-domain antibodies for the treatment of adult patients affected by R/R MM were published [80]. Interestingly, the ORR was 97.9% (the total number of enrolled patients = 97), with most patients (83%) achieving a complete response at 27.7 month follow-up. Among those patients, the CRS occurred in 92 patients (95%), with only 5% of Grade 3 or higher. Furthermore, ICANS was observed in 16% of the patients (mainly Grade 1–2). In 2022, the FDA approved the ciltacabtagene autoleucel immunotherapy for the treatment of adult patients with R/R MM after four or more prior lines of therapy [81].

## 4. CAR-T Cells in Solid Tumors

Given that CAR-T-based therapies have been used successfully to treat B cell malignancies [82], this has generated great interest in the extension of the CAR-T cell approach to treat solid tumors. Nevertheless, variable or low efficacy in this context has been demonstrated so far [83]. This is mainly associated with the nature of these malignancies, including the presence of important barriers that can prevent the entry and persistence of CAR-T and other effector cells of our immune system, the hostile microenvironment, as well as the tumor heterogeneity [84,85,86]. Therefore, a close association between the specificity of the CAR and its safety, and its efficacy may be considered when new CAR-T cells are developed against solid tumors [87,88,89].

Besides well documented side effects, CAR-T cells have attracted great interest for the treatment of tumors of different origins.

### 4.1. Ovarian Cancer

Relevant efforts in the study of CAR-T cell efficacy have been focused on ovarian cancer. Tumor-associated glycoprotein 72 (TAG72), being highly expressed on the ovarian cancer cell surface, has been used as a target of CAR-T cell therapy in such a context. Those CAR-T cells demonstrated significant cytotoxic functions and relevant cytokine production, increasing the lifespan of treated mice [90]. It has been shown that CAR-T cells that were developed using MUC16 as a CAR induce the inhibition of ovarian cancer progression and the suppression of malignant cells [91]. Therefore, those CAR-T cells have been proposed for ovarian cancer treatment [91]. It has been also demonstrated that the growth of SKOV3 cells that express human epidermal growth factor receptor 2 (Her2)/neu was suppressed by Her2 CAR-T cells [91]. Additional CARs, such as 5T4 and FRα, were successfully used against ovarian cancer cells [92,93].

### 4.2. Pancreatic Cancer

Pancreatic cancer (PC) is characterized by a heterogeneous microenvironment, contributing to disease progression and resistance to chemotherapy. Many target candidates for CAR-T cell therapies against PC are currently under investigation in several clinical trials, among them are mucin 1 (MUC1), Claudin 18.2, prostate stem cell antigen (PSCA), prominin 1 (PROM1), epidermal growth factor receptor (EGFR), and mesothelin (MSLN) [94]. In particular, MSLN was largely studied as a target candidate, given that it is a cell surface glycoprotein that is expressed on nearly all PDAC tumors (85–100%), with low expression on mesothelial cells in the pleura, peritoneum, and pericardium [95]. It is known that the efficacy of anti-mesothelin CAR-T cells is dependent on the abundance of mesothelin epitopes [96]. Those CAR-T cells were effective in vitro, against mesothelin-positive cells, and also in vivo in immunodeficient mice with ovarian cancer-expressing mesothelin (OVCAR-8), in pancreatic cancer (KLM-1), and in patient-derived tumor mesothelioma xenografts (NCI-Meso63) [96]. Recently, CEACAM7 has been identified as a new target for PC treatment due to its relevant surface expression on primary human PC tumor cells, as compared to its poor expression in normal tissues [97]. In addition, CEACAM6, together with CD318 and TSPAN8, were identified as promising targets for CAR-T cell immunotherapy against PC [94].

However, even though important results were observed in preclinical studies, MSLN-CAR clinical trials showed low anti-tumor efficacy, possibly due to the heterogeneity of MSLN expression on tumor cells, as well as limited tumor penetration, or the lack of T cell persistence and exhaustion. Instead, CAR-T cells targeting CEACAM7 show significant remission of late-stage patient-derived PC xenograft tumors. Due to its restricted tissue expression, CEACAM7-directed CAR-T cells may offer a higher safety margin than commonly used targets such as CEACAM5 and HER2 with broader systemic expression [97].

### 4.3. Breast Cancer

Breast cancer cells are characterized by the modified expressions of several molecules that could be candidates as potential targets for CAR-T cell therapies. In a Phase I study (NCT03545815), it was shown that the combined administration of MSLN CAR-T, together with programmed cell death protein 1 (PD-1) blockade, strongly increased the cytotoxicity and persistence of T cells within the tumor [98,99]. In addition, it is reported that the use of epithelial cell adhesion molecule, EpCAM CAR-T cells, could foster cytokine release (i.e.,: interferon-γ, IL-2, and IL-6), which in turn induces strong apoptotic effects on cancer cells [100].

### 4.4. Hepatocellular Carcinoma

Hepatocellular carcinoma (HCC) is the most common liver cancer, and its incidence and mortality rates are overlapping. Multitargeted CAR-T cells have been developed against HCC, and among them, Glypican-3 (GPC3), alpha-fetoprotein (AFP), c-MET, and Mucin 1 NKG2DL have been used as targets to develop CAR-T cells [101,102,103]. Interestingly, it was demonstrated that second and third-generation GPC3 CAR-T were effective against HepG2 and Huh-7 tumor cell lines, both in vitro and in vivo [101]. Therefore, two different Phase I trials using autologous second generation 41BBζ GPC3 CAR-T in adults with advanced HCC were performed [NCT 02395250; NCT03146234], and those studies are included in the 22 clinical trials investigating the use of CAR-T for HCC treatment.

### 4.5. Glioblastoma

Glioblastoma (GBM) is the most common and aggressive malignant primary brain tumor in adults. Current treatment options usually involve surgery followed by chemotherapy or radiotherapy; however, life expectancy still remains extremely short (one year) [104]. Similar to other solid tumors, CAR-T cell therapies against GBM still face several challenges, such as tumor heterogeneity, tumor immunosuppressive microenvironment, and CAR-T cell homing and persistence. Several GBM-specific targets have already been identified including EGFRvIII, HER2, erythropoietin-producing hepatocellular carcinoma A2 (EphA2), GD2, or IL-13Rα2 [105,106,107,108,109]. Variant III (EGFRvIII) is the most common gene mutation of epidermal growth factor receptor (EGFR) and it is present in about 52% of glioma cells, but not in normal tissues [105]. A clinical study of CAR-T-targeting EGFRvIII was effective against GBM cells, showing, however, a dose- and time-dependent release of cytokines, with a relevant induced glioma cell toxicity in patients with recurrent GBM [105]. The same study also demonstrated that CAR-T cells induced the antigenic loss, making the tumor resistant to the treatment. More recently, a bold trivalent CAR strategy using a single lentiviral construct expressing three individual CARs directed against IL-13Rα2, HER, and EphA2 was developed to address the glioma antigenic escape issue [105]. Notably, IL13Rα2 is commonly expressed in more than 75% of GBMs, playing a crucial role in GBM invasiveness and progression [110]. As a matter of fact, human IL13Rα2 CAR-T therapy improves the GBM immune microenvironment and induces the activation of host immune cells [111]. The potential of HER2 as a new target for GBM has been also investigated, given that HER2 is overexpressed in GBM, as in many other human cancer types [112]. Furthermore, an ongoing clinical trial is studying the safety and efficacy of CAR-T cells targeting HER2 in subjects with progressive recurrent or refractory HER2-positive primary central nervous system (CNS) tumors or HER2-positive tumor metastatic to the CNS after standard intervention (NCT02442297).

### 4.6. Prostate Cancer

Prostate cancer (PCa) represents the second most frequently diagnosed malignancy among all male malignant tumors [113]. There are three main targets of CAR-T therapy for treating prostate tumors that have been studied, including prostate-specific membrane antigen (PSMA), PSCA, and EpCAM [114]. Among them, it is known that PSMA is overexpressed in prostate cancer and the endothelium of tumor neo-vasculature, even if its expression was also evidenced in many normal tissues [115]. It has been also shown that CAR-T cells targeting PSMA display significant anti-tumor effects on PSMA-positive tumor cells in vitro and in vivo [116]. Recently, studies that were carried out on prostate cancer cells using second-generation anti-PSMA CAR-T cells exhibited high cytotoxicity in in vitro models, even if the same results were not obtained when they were used in vivo. This lack of in vivo efficacy might be due to the effects that were exerted by the dynamic inhibitory tumor microenvironment. Furthermore, prostate stem cell antigen (PSCA) is a tumor-related molecule that is expressed in 90% of prostate cancer tissues, but also in 60–70% of normal prostate tissues. Currently, two Phase I/II clinical trials are underway to evaluate the efficacy and safety of these CAR-T cells targeting PSCA in patients with advanced prostate cancer (NCT03873805, NCT02744287) [114]. In addition, EpCAM is another antigen that is expressed by many types of human epithelial carcinomas, such as lung, breast, and prostate. However, even if the expression of EpCAM in prostate cancer is variable, one clinical trial evaluating the safety and efficacy of EpCAM CAR-T cells in patients with EpCAM-positive cancer is ongoing (NCT03013712) [114].

### 4.7. Renal Cancer

It has been reported that the antigen carboxy-anhydrase-IX (CA-IX), a metalloprotease that is involved in carbon dioxide hydration, is expressed by different types of renal cancers. Therefore it has been proposed as a novel CAR-T cell therapy target [117,118]. It is also known that CA-IX is expressed by several normal tissues (i.e., small intestine epithelium, gastric mucosa epithelium, duodenum, and biliary tree), but with low/moderate expression [119]. Notably, the expression of CA-IX may increase in a wide range of tissues under hypoxic conditions [120]. At present, a first generation of CA-IX CAR-T cells have been produced and they demonstrated cytotoxic functions [121].

### 4.8. Gastric Cancer

Bi-specific Trop2/PD-L1 CAR-T cells have been developed to fight gastric cancer cells. These CAR-T cells appeared able to induce the inhibition of gastric cancer growth upon their intra-tumoral injection [122]. It has been also demonstrated that MSLN CAR-T cells are effective in inducing gastric cancer cell death and inhibition of tumor growth [123]. Other antigens have been targeted with CAR-T cells in gastric cancer, including folate receptor 1 (FOLR1), claudin 18.2, HER2, and NKG2D [124,125,126,127]. More recently, it has been shown that ICAM-1 CAR-T cells alone or in combination with CAR activation-dependent interleukin (IL)-12 release or paclitaxel, improve the outcome of advanced gastric cancer patients expressing ICAM-1 [128].

### 4.9. Colorectal Cancer

It is known that colorectal cancer cells prominently express CD133, TAG-72, NKG2D, and Guanylate Cyclase 2C, and specific CAR-T cells redirected versus these antigens have been generated [89,129,130,131]. Recently, it has been demonstrated that mesenchymal stem cells (MSCs) engineered to release IL-7 and IL-12 increase the anti-tumor activity of CAR-T cells with specificity for the carcinoembryonic antigen (CEA), acting against colorectal carcinoma cells and altering the inflammatory action of Th2 in the tumor milieu [132]. CAR-T cells using DCLK1 as a target are also effective against primary and metastatic colon cancer cells [133].

### 4.10. Lung Cancer

Many different studies have demonstrated that specific CAR-T cells can be effective against lung cancer. CAR-T cells directed against the receptor tyrosine kinase-like orphan receptor 1 (ROR1) showed a strong anti-tumor activity in human lung cancer A549 cell lines. It has been demonstrated that those ROR1 CAR-T cells are able to infiltrate the tumor tissues and eradicate several layers of tumor cells [134]. Moreover, EGFRvIII CAR-T cells have been produced and tested. Those cells strongly kill A549-EGFRvIII cells through the expression of some key molecules, such as TNF-α, IFN-γ, granzyme B, and perforin. Notably, EGFRvIII CAR-T cells were able to reduce the A549-EGFRvIII cell metastasis in mice, with a significant extension of mouse survival without any side effects [135]. Some other molecules have been successfully used as CARs to treat lung cancer. Among them, EphA2, MSLN, mucin-1, and PSCA must be cited [136,137,138]. CAR-T cells using PD-L1 as a CAR against non-small cell lung carcinoma (NSCLC) exerted anti-tumor cytotoxic effects against PD-L1-high and EGFR-mut NSCLC, leading to the recovery of PD-L1+ NSCLC patients [139].

Regarding small cell lung cancer treatment, an attractive target is represented also by delta-like 3 (DLL3). It has been shown that DLL3 CAR-T cells alone or in combination with PD-1 inhibitors are able to kill DLL3+ tumor cells [140].

**Figure 2:**
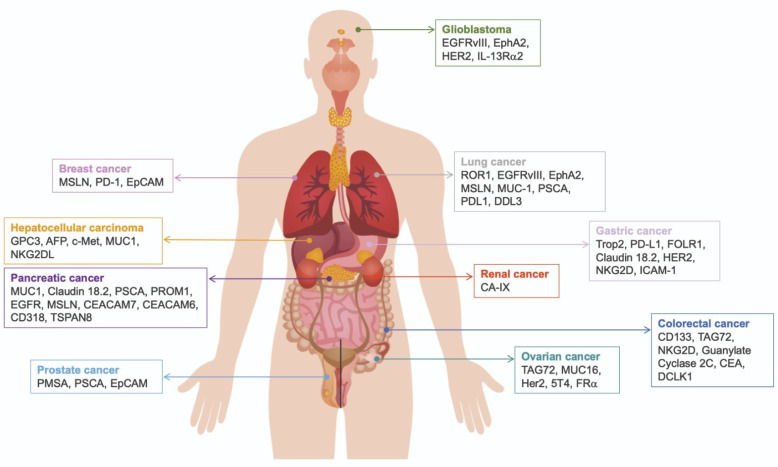
Schematic representation of the targets used to produce CAR-T cells to treat solid tumors.

All the identified and tested CARs to produce CAR T cells against solid tumors are summarized in Table 1 and Figure 2.

## 5. Structure and Biogenesis of Extracellular Vesicles

**Figure 3:**
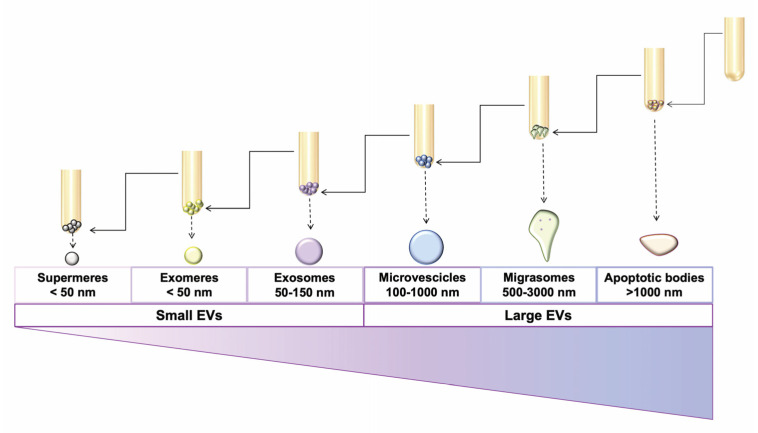
Schematic representation of extracellular vesicle subtypes. Large extracellular vesicles include EV subtypes with diameters larger than 100–200 nm, such as apoptotic bodies, migrasomes, and microvescicles. Small extracellular vesicles include exosomes, exomeres, and supermeres (supernatant of exomeres), all characterized by diameters smaller than 200 nm. In some cases, large and small EV size overlap.

Conventionally, EVs have been classified based on their biogenesis processes that, in turn, tends to affect their dimension [144,145]. There are three main EV subtypes that are usually described: exosomes, microvesicles, and apoptotic bodies. More recently, additional morphologically and structurally distinct EV subtypes have been described; among them “exomeres” that are large macromolecular complexes and small non-membrane- bound nanoparticles with diameters smaller than 50 nm, have been included under the “EV” umbrella term [146]; supermeres (a morphologically distinct EV population displaying an in vivo markedly greater uptake compared with small extracellular vesicles and exomeres) [147]; and migrasomes (vesicular structures that mediate a cell migration mechanism called migracytocis) [148] have been also identified (Figure 3). Little is known about these newly described EV subtypes and they are still not included in the most recent ISEV MISEV guidelines. Conversely, exosomes, microvesicles, and apoptotic bodies have been largely studied.

Exosomes, the smallest EV subtype, display diameters ranging from 30–50 to 150 nm [149,150]. They are released by the fusion of multivesicular endosomes or bodies (MVE or MVB) with the plasma membrane [151,152], either through mechanisms that are dependent on the proteins of the “endosomal sorting complex required for transport” (ESCRT) or ESCRT-independent [153,154]. The sorting of the cargo within the exosome, which can be itself either ESCR- dependent or ESCRT-independent, is apparently highly specific and it is drowned by the formation of membrane microdomains, which are generated by the clustering of the lipids and the membrane-associated proteins. These membrane microdomains participate in the recruitment of soluble molecules, such as RNAs and cytosolic proteins that will be further sorted within exosomes [2]. In more detail, in the ESCRT-dependent pathway, the ESCRT machinery involves, as a first step, the recruitment of the ESCRT-0 and ESCRT-I subunits that, at the limit of MVB membranes, induce the clustering of ubiquitylated transmembrane cargoes at the microdomain levels. The ESCRT-II is further recruited to induce the budding and the fission of these microdomain-cargo clusters [155]. The inhibition of the ESCRT components affects the exosome release and/or the sorting of their cargoes [156]. As mentioned, the exosome release may also be drawn by an ESCRT-independent pathway, given that it has been also demonstrated that the formation of the MBVs persist even upon the depletion of four ESCRT complexes [157]. Among the ESCRT-independent mechanisms of exosome biogenesis, the pathway based on the ceramide presence must be cited. In this context, the ceramide presence is associated with the formation of membrane subdomains, through the intervention of the neutral Type II sphingomyelinase that is able to induce the hydrolysis of sphingomyelin to ceramide [158]. Furthermore, the proteins of the tetraspanin family have been also involved in the ESCRT-independent endosomal sorting. Among tetraspanins, CD9, CD81, and CD82 have been associated with the cargo sorting of exosomes [159,160], while CD63 has been involved in the endosomal sorting processes that are associated with the genesis of exosomes from the melanocytes, melanoma cells, and fibroblasts of patients affected by the Down syndrome [154,161,162,163]. If the exosome biogenesis is drawn by ESCRT-dependent or ESCRT-independent mechanisms, this could depend on the parental cell releasing the exosomes or on the cargo within the generating exosomes [159,160].

Microvesicles (or microparticles), displaying diameters ranging from 100 to 1000 nm, are directly released by the budding of the plasma membranes, and for these reasons, they are also known as shedding vesicles, ectovesicles, or ectosomes [164].

Apoptotic bodies are the largest EVs (0.1–5 µm) that are released during the latest apoptosis stages [165,166], exposing the phosphatidylserine and carrying caspases 3 and 7 and their substrates (ROCK1 and PANX1) [167].

However, more recently, the International Society of Extracellular Vesicles (ISEV) established that the aforementioned classification produced confusion, given that many recent findings have demonstrated that EV subtypes overlap in diameters, as well as in functions and cargoes [168]. For these reasons, the ISEV recommended the use of the “extracellular vesicles” umbrella term for all EV subtypes, identifying small EVs as those with diameters smaller than 200 nm and medium/large as the EVs with diameters larger than 200 nm [169].

It is known that EVs are released in physiological and pathophysiological conditions, and their characteristics are influenced by cell topography, environmental factors, and external stimuli [170,171].

For these reasons, body fluids are a heterogeneous mixture of EVs that are released by different parental cells. In peripheral blood samples, EVs stemming from leukocytes, erythrocytes, platelets, endothelial cells, and even from cancer stem cells in cancer patients have been described [13,14,20,21,22,23,172,173].

It has been also demonstrated that EVs carry specific biomolecules that are related to the features, the functions, and the actual status of their parental cells. For these reasons, by studying their phenotypes, it is possible to identify the compartment from which they originated [14,17,169,172,174,175]. Many EV phenotypes have been identified in body fluids as well as in cell culture media [14,176]. For all these reasons, EVs dynamically reflect the actual status of any compartment of the body, therefore, they have been proposed as reliable biomarkers for liquid biopsy [11,173,177,178,179,180]. On the other hand, EVs are emerging as signalosome elements that are able to mediate adaptive responses, locally and systemically, with relevant implications in physiological and pathophysiological events in vivo [10]. These functions are determined by the EV cargoes and are addressed to target cells by specific surface-exposed ligands and receptors, and able to mediate the EV binding to target cells or to the extracellular matrix. Such an interaction with target cells or EV internalization further triggers intracellular signaling pathways within the recipient cells [10]. Moreover, EVs change target cell phenotypes, transferring active receptors, such as EGFRvIII and CCR5 [181,182]. The EV uptake by the recipient cells may occur through phagocytosis or membrane fusion [183,184]. EVs transfer their cargoes to the target cells through micropinocytosis [185,186,187]. It has been also shown that the delivering of molecules by the EVs are more effective on target cells than the corresponding soluble form of the same molecule, given that plasma membranes protect EV cargoes from the circulating enzyme degradation activities [188,189]. The interaction with the target cell produces different effects that are related to the phenotype and to the features of their parental cells, as well as to the mechanisms that induced EV release [10].

In addition to phagocytosis, membrane fusion, and micropinocytosis, there are a variety of ways that are used by EVs to interact with the recipient cells, such as macropinocytosis and lipid-raft-mediated uptake [2,190,191]. It has been reported that EVs also use the cargo transfer that is mediated by tunnelling nanotubes to modify the biology of target cells [192]. The clathrin/caveolin-mediated endocytosis is the most used process of EV internalization by target cells [2,190]. When delivered within the target cell, the EV cargo exits from the endosome pathway into the cell’s cytoplasm before the endosome’s maturation and acidification, avoiding consequent degradation [193]. This escape from the endosomal pathway is crucial for functional delivery of the EV content [194]. It is currently unknown whether EVs have evolved unique methods or mechanisms similar to viruses and bacteria to escape from this fate [195]. However, the EV cargo can be degraded, recycled within the cell, or re-released as intact vesicles into the extracellular space. It has also been established that the pattern of EV surface proteins is strictly related to their cargo fate [183,196,197,198]. In any case, the EV uptake often leads to lysosomal degradation for the recycling or the elimination of molecules from the recipient cells [199]. Using these mechanisms, EVs transfer many different types of biomolecules to target cells, including nucleic acids, proteins, and lipids. The subcellular localization of cargo molecules delivered by EVs and the compartments where these molecules carry out their functions depending on the cargo type and the target cell. The direct investigation of the fate of EV cargo within intracellular exosomes is a technically challenging task and many studies typically employ the labelling of EV membrane lipids and proteins to this end. Despite recent progress in understanding the efficiency of cargo loading, the direct evidence of cargo release is still limited [200].

## 6. T-Lymphocytes-Derived Extracellular Vesicles

Extracellular vesicles that are secreted by T-lymphocytes modulate the activity of immune responses regulating the activity of immune cells [201]. Leukocyte-derived EVs have been implicated in many immune response functions, such as the recognition and the removal of pathogens and harmful substances, and inflammatory responses [202,203]. In such a context, great interest has been focused on T-lymphocyte-derived EVs. Several T cell subsets, such as cytotoxic T-lymphocytes (CTL), T helper (Th), and T regulatory cells (Tregs) may release active EVs within the extracellular milieu [187,204]. Stimulation conditions (i.e., the presence of co-stimulatory molecules) participate in the regulation of the release of different EV subtypes [205]. Although a large proportion of cargo proteins is also common in EVs that are produced by other cell types [206,207], they express specific T cell receptors and adhesion molecules [201,207]. The study of EVs stemming from T-lymphocytes revealed that they express different typical markers. As described below, the release of EVs from T-lymphocytes is highly influenced by lipid metabolism and signaling. For instance, silencing nSMase2, or inhibiting its activity, produces a significant decrease of ceramide production with a consequent inhibition of EV release by T cells [208]. Furthermore, aSMase plays a pivotal role in the secretion mechanisms of CTL-derived EVs [209]. In addition, diacylglycerol kinase α (DGKα), lipid kinase that controls T cell activation, has been involved in EV secretion by T-lymphocytes [210,211,212,213], and its inhibition increases EV release [211,212].

It was shown that EVs that are released from Tregs inhibit CD8+ T cell responses [214]. Furthermore, Treg-derived EVs modify dendritic functions, induce an increase in IL-10, and a decrease in IL-6 production, participating in the inhibition of immune reaction in tissues through the transfer of miR-150-5p and miR-142-3p [215].

It is worth noting that the activation of T-lymphocytes induces the release of EVs with proapoptotic functions due to FasL and Apo2 expression [216]. FasL and Apo2L on EV surfaces bind to target cells, interacting with their respective receptors, thus inducing apoptosis through the caspase pathway activation [217]. It was also shown that EVs that are released by Th cells, enriched in TCR, may participate with the immunological synapses that are formed by B lymphocytes that act as antigen presenting cells, stimulating pMHC-II signaling [218,219]. Furthermore, CTL-derived EVs express CD3, CD8, and TCR [212,220,221]. It is also interesting to note that EVs that are released by CTL and natural killer cells (NK) express some other functional molecules, such as perforin and granzyme A and B [222].

**Figure 4:**
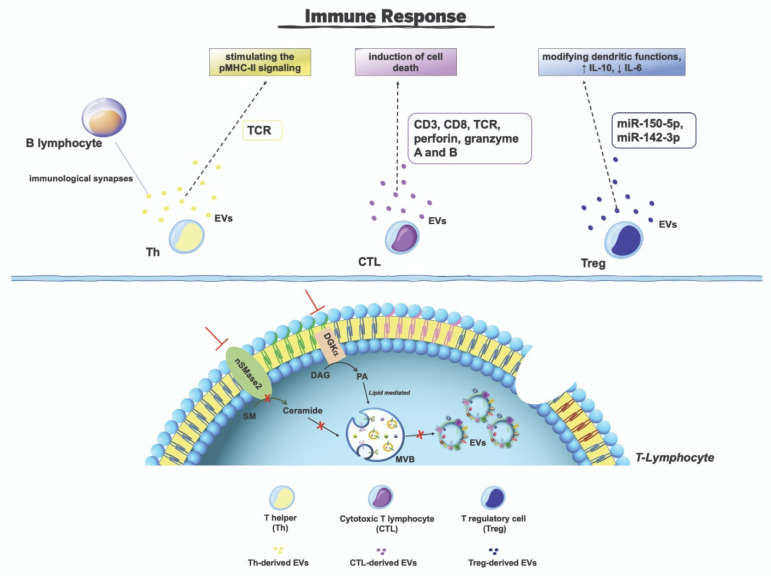
T-lymphocyte-derived extracellular vesicle involvement in immune responses.

An RFA schematic representation of immune functions of T-lymphocyte-derived EVs is shown in Figure 4.

## 7. Chimeric Antigen Receptor (CAR)-T Cell-Derived Extracellular Vesicles

CAR-T EVs present many potential advantages when compared with the CAR-T parental cells. First, CAR-T EVs are stable, but they have a limited life span and are incapable of proliferating, designating an immunotherapy product that is associated with low collateral toxicity risks (e.g.,: low incidence of CRS) [143]. In such a context, it is known that CAR-T-induced CRS, an uncontrolled release of cytokines by CAR-T cells that is one of the most serious and frequent complications that is associated with CAR-T cell infusion, occurring approximately 10 days after the CAR-T cell administration [223]. In line with this lower toxicity potential, CAR-T EVs can be used as cell-free immunotherapy agents and, therefore, they should not be considered advance therapy medicinal products, which may simplify regulatory agency approval. Furthermore, considering their low immunogenicity in heterologous infusions, it is conceivable that they might be used in a third-party setting as off-the-shelf product. Then, differently from the parental cells, EVs may easily cross tumor-barriers, as demonstrated by the presence of tumor-derived EVs in body fluids [172,223], even in tumors that are characterized by strong fibrotic reaction [23].

Some studies also underlined how cell-free EV therapies could be implemented in terms of EV production and functionality by appropriately stimulating the producer cells [143]. Lastly, in the case of autologous CAR production from leukemia patients with circulating disease, CAR-T EV therapies may have the advantage of not incurring the risk of re-infusing tumor cells into the patient.

In this regard, it has been reported that residual tumor cells may be incidentally transduced with CAR, which may lead to CAR-T cell therapy resistance [71].

The expression of TCR and proapoptotic molecules (granzymes A and B, perforin, Apo2L, and FasL) by EVs that are derived from T-lymphocytes confer them cytotoxic functions and antigen specificity [220,222]. For these reasons, it was hypothesized that CAR-T cell-derived EVs might be potent vectors delivering proapoptotic messages to cancer cells. However, the surface expression of the CAR molecule on CAR-T cell-derived EVs is mandatory to specifically induce cancer cell death as it occurs for their parental CAR-T cells [82,223].

The production of EVs expressing the CAR can be optimized both in terms of increase of EV release and induction of CAR expression on EV surfaces. In more detail, it is known that TCR activation boosts the production of CTL-derived EVs [221], while higher levels of CAR expression in Evs can be obtained using different antigen stimulation strategies, such as cells beads that are coated with the recombinant CAR target antigen, or cells expressing the CAR antigen [143].

A carefully designed proof-of-principle study demonstrated that CAR-T EVs with EGFR and HER-2 specific CAR efficiently and specifically kill HER2+ and EGFR+ cancer cells in mouse xenograft models, without impacting cells that do not express those molecules [143]. Moreover, in that study, PD-L1 was shown to inhibit CAR-T cells, but not CAR-T EVs both in vitro and in vivo, suggesting a better resilience of EVs to tumor immune evasion strategies. Finally, the same study demonstrated that differently from CAR-T cells, CAR-T EVs did not elicit cytokine release syndrome in mouse models [143]. Altogether high target specificity, insensitivity to PD-L1 immunosuppression, and a lack of cytokine release syndrome induction appear promising advantages of CAR-T EVs on their cellular counterpart. Similar results have been obtained using EVs that are derived from mesothelin (MSLN)-targeted CAR-T cells, showing their efficiency in targeting MSLN-positive and triple-negative breast cancer cells via secretion of perforin and granzyme B. More importantly, the study demonstrated a marked antitumor effect with low toxicity in vivo, in both BT-549 and MDA231-MSLN xenograft breast tumor models [142].

Recently, it was shown that CAR-T carrying a CAR construct driving the transcription of RN7SL1, a non-coding RNA that induces the stimulation of IFN genes, secrete EVs delivering RN7LS1 [224]. Those CAR-T EVs improve immune activation against tumor cells, transferring RN7SL1 to endogenous immune cells, but not to tumor cells. RN7SL1 recipient cells, which are T, myeloid, and dendritic cells, activate IFN-dependent inflammatory responses, improving the immunostimulatory effects of dendritic and myeloid cells that end up in activation of CD8 T cells against tumor cells. These activated immune cells, in turn, orchestrate solid tumor rejection, even when tumors lack adequate expression of neoantigens [224]. Therefore, these data show that RN7SL1-containing CAR-T EVs synergize with immune cells to increase their efficacy against cancer cells.

In summary, the use of EVs derived from CAR-T cells to treat solid tumors may allow overcoming of some issues that are related to CAR T cell treatments with consequent advantages, such as the ability of EVs to easily cross the physical tumor barriers and to avoid the negative impact of the immunosuppressive tumor microenvironment that largely alters CAR-T cell functionality [35,36,37,38]. Furthermore, due to their limited life span, their inability to proliferate, and possibly, to their lower content of cytokines related to CRS and to neurotoxicity, as well as to their slower induction of apoptotic effects [141], EVs were shown to be associated to lower collateral toxicity risks as compared to CAR-T cells (e.g., low incidence of CRS) [143]. In addition, EVs are not considered advanced therapy medicinal products, which may simplify regulatory agency approval. Of note, considering their low immunogenicity in heterologous infusions it is conceivable that they might be used in a third-party setting as off-the-shelf products. Finally, CAR-T EV therapies may have the advantage of not incurring the risk of re-infusing tumor cells into the patient, given that it has been demonstrated that, together with CAR-T, residual tumor cells may be incidentally transduced with the CAR, which may lead to CAR-T cell therapy resistance [71].

An intriguing extension of this field of research concerns the antitumor potential of CAR-NK-derived EVs. Considering that CTL and NK cells use the same cytotoxic effectors (i.e., Fas L, granzymes, and perforins) [225,226,227,228], it may be speculated that also CAR-NK EVs might have a great potential as therapeutic agents. Furthermore, in vitro NK cell expansion increases EV release [229], as also demonstrated for T cells, which may provide an efficient strategy to produce Evs on large scale, particularly useful for EV preclinical and clinical applications [229]. However, NK cell recognition of targets is not dependent on antigen specificity, but it is related to the integration of different signals that are associated with many NK receptors, requiring a deep analysis of NK functions before planning any experimental design. This is probably the leading cause of the scarce development of CAR-NK, which may also interfere with the development of CAR-NK EVs therapies [229,230]. Nevertheless, given that the use of CAR-NK cells presents some advantages such as greater safety and more efficient EV production, both CAR-NKs and CAR-NK EVs appear interesting fields of research for the development of safer and more efficient antitumor therapeutic strategies.

## 8. Extracellular Vesicles as Drug Delivery Systems

EVs share many characteristics with synthetic nanocarriers. Both systems allow proper drug delivery by controlling the space and the time distribution of drugs, minimizing the related off-target side toxicity. The optimization of drug delivery systems (DDSs) has greatly advanced over the last few decades and sophisticated synthetic nanocarriers have been engineered to resemble biological structures and improve the circulation, targeting, and responsiveness of a wide range of therapeutics including small molecules, peptides and nucleic acids. Even if they display some advantages related to low production costs, easy upscale production, and favorable physico-chemical properties in terms of shape, size, and surface properties, they suffer from a poor in vitro-in vivo correlation (IVIVC) and unpredictable nano-bio interactions [231,232]. Starting from this evidence, published data suggest that there are still many issues in the drug delivery field for a satisfactory targeting specificity. These challenges related to the physical interactions with different biological environments may be overcome by biological or bio-derived drug delivery systems. Cell-derived EV-based carrier systems are well integrated in this frame. They combine the unique functionalities of natural materials and multifunctional design of synthetic nanomaterials. Since they are generated by an endogenous source, the natural cargo (miRNA, DNA, proteins, and lipids) composition, and their natural involvement in the intercellular communication, they have gained considerable interest and were proposed as a novel generation of delivery systems, potentially useful for solving the well-known unsatisfactory response to conventional treatments. It has been reported that EVs, due to their inherent targeting characteristics, are able to establish advantageous interactions with target cells more than liposomes, the synthetic homologous nanostructures [233].

Indeed, given that EVs home target tissues crossing the biological barriers, the EV drug delivery system, together with their pharmacokinetic profile, may result in a higher therapeutic efficacy when compared to synthetic liposomes. In such a context, the biodistribution of EVs is crucial both in terms of efficacy and safety, and, for these reasons, it was analyzed using different mouse models, using fluorescent labeled EVs, or luciferase tagged vesicles. In this way it has been demonstrated that EVs accumulate in the liver, spleen, kidney, and in gastrointestinal tract and that the biodistribution could be influenced by the route of administration, and by the EV origin and size [234,235,236]. On the other hand, it is known that liver and spleen are the main accumulation organs of liposomes.

Moreover, EVs have been further engineered to improve their delivery capabilities. They have been modified by introducing specific ligands, stimuli-responsive moieties, and immune evasive factors, resulting in carrier systems which simultaneously take advantages from biological and exogenous features. In a recent study, different cell types were genetically engineered to enrich specific siRNAs in EVs, improving the functionality of in vivo siRNA delivery, significantly reducing siRNA therapeutic doses with respect to those typically that are delivered via lipid nanoparticles [237]. Despite the EVs advantages in targeting, safety, and pharmacokinetics over the synthetic nanocarriers, many challenges that are related to the upscaling production, isolation, purification, characterization, and storage, need to be faced for EV clinical translation [238].

The above-mentioned systems are classified as biological and chemical methods and refer to carrier-mediated strategies. Further approaches that are used for the intracellular delivery of cargoes include physical techniques based on a membrane-disruption-mediated mechanism. Usually, the carried-based delivery exploits a fusion or endocytic entry route, while the membrane disruption approaches involve different entry pathways including permeabilization and direct penetration processes.

EVs have shown great potential for the intracellular delivery of different cargoes including both synthetic and biological molecules. Examples of loading mechanisms, applicable in a post-EV isolation stage mix electroporation, sonication, cycles of freeze-thaw, extrusion, and the employment of pore forming agents. It is important to highlight that the above-mentioned techniques are useful for improving the loading, but they may be responsible for different negative effects that result in a loss of the EV membrane integrity, as well as aggregate production and cargo impurity. Haney et al. observed that, for protein catalase, the loading efficiency decreased in the following order: sonication > extrusion > surfactant treatment > freeze-thaw treatment > incubation, with the higher and the lower values of 26 ± 1.2% and 4.9 ± 0.5%, respectively. Similarly, in other studies, sonication, compared to other methods, guaranteed the higher loading of chemotherapeutics and hollow gold nanomaterials [239]. The exosomes cargo loading efficiency is limited when compared to synthetic liposomes [240]. Nevertheless, many investigations are focused on the technical optimization of these loading procedures, even if guidelines for the EV loading and standardized procedures are still lacking. Indeed, EV heterogeneity hampers the evaluation of the loading performance through the entrapment efficiency and loading capacity, parameters that are commonly used to assess the suitability of a drug-carrier system [241,242].

The generation of an off-the-shelf EV-based product is emerging as a new frontier of drug delivery systems. Furthermore, EV stability remains the major issue for their implementation in clinical settings. Interestingly, Görgens et al. recently carried out an in-depth analysis of EV stability on different storage buffer formulations, studying how they affect their surface integrity and biological activity. EVs that were obtained from cell culture supernatants were stored up to 2 years at different temperatures and with different methods. They found that a freezing solution containing the HEPES buffer, albumin, and trehalose obtained the best EV preservation for long-term storage and for other downstream analyses even with several freeze-thaw cycles [243].

## 9. Conclusions

The application of CAR-T Evs as therapeutic agents might allow us to obtain a cell-free product that possibly results in more controllable adverse effects. It could also prevent the CRS syndrome onset and related complications. Furthermore, the use of CAR-T Evs may improve the traffic of CAR-T effector functions by the infiltration of solid tumors, given that Evs may more easily cross physical tumor barriers. Furthermore, even if some recent reports demonstrated that EV treatments should be added to CAR-T cell-based therapies [142], further studies are needed to clarify which scheme is more suitable between the substitution of CAR-T cell-based therapies with CAR-T Evs or the complementary use of CAR-T together with their Evs. Strategies to optimize the in vitro production of bioactive CAR-T Evs are also needed. Finally, strategies to increase the EV homing efficiency to their specific targets must be optimized to reduce the EV dispersion.

## Figures and Tables

**Table 1:** List of targets used to produce CAR-T cells to treat solid tumors.

Cancer Type	Targets Used for CAR-T Cell Production
Ovarian Cancer	TAG72 [90]; MUC16 [91]; HER2 * [91] [141]; 5T4 [92]; FRα [93]
Pancreatic Cancer	MSLN [95,96]; CEACAM7 [97]; CEACAM6, CD318, TSPAN8 [94]
Breast Cancer	MSLN * [98,99,142]; EpCAM [100]; HER2 * [141,143];
Hepatocellular Carcinoma	GPC3 [101]; AFP [102]; NKG2DL [103]
Glioblastoma	IL13Rα2 [110,111]; HER2 [112]; EphA2 [106,107]; EGFRvIII [105]
Prostate Cancer	PSMA [115,116]; PSCA [114]; EpCAM [114]
Renal Cancer	CA-IX [121]
Gastric Cancer	Trop2/PD-L1 [122]; MSLN [123]; FOLR1 [126]; claudin 18.2 [124]; HER2 [127]; NKG2D [125]; ICAM-1 [128]
Colorectal Cancer	CD133 [130]; TAG-72 [131]; NKG2D [129]; Guanylate Cyclase 2C [89]; CEA [132]; DCLK1 [133]
Lung Cancer	ROR1 [134]; EGFRvIII [135]; EphA2 [137]; MSLN [136]; mucin-1 [138]; PSCA [138]; PD-L1 [139]; DLL3 [140]

* Common targets between CAR-T cells and CAR-T-derived EVs used in preclinical studies.
